# A multisensory mindfulness experience: exploring the promotion of sensory awareness as a mindfulness practice

**DOI:** 10.3389/fpsyg.2023.1230832

**Published:** 2023-11-09

**Authors:** Carolyn Finck, Alba Avila, William Jiménez-Leal, Juan Pablo Botero, Daniel Shambo, Susana Hernandez, Felipe Reinoso-Carvalho, Veneta Andonova

**Affiliations:** ^1^Department of Psychology, Faculty of Social Sciences, University of Los Andes, Bogotá, Colombia; ^2^Department of Electrical and Electronics Engineering, Faculty of Engineering, University of Los Andes, Bogotá, Colombia; ^3^Center for Microelectronics (CMUA), Faculty of Engenieering, University of Los Andes, Bogotá, Colombia; ^4^Department of Biomedical Engineering, College of Engineering, University of Utah, Salt Lake City, UT, United States; ^5^School of Management, University of Los Andes, Bogotá, Colombia

**Keywords:** mindfulness, sensory stimulation, technology, heart rate variability, biofeedback, multisensory experience design

## Abstract

**Objectives:**

In this preliminary and multidisciplinary exploratory study, we assessed whether a mindfulness practice could be enhanced through a multisensory experience design that mimics the “beginner’s mind,” relying on sensory awareness and biofeedback processes as participants interact with the experience.

**Methods:**

We piloted and designed two conditions, being (a) a guided mindfulness practice based on the senses as an anchor to the present moment, using audio instruction only; and (b) an experience of mindfulness practice with successive sensory stimulation (olfactory, audio, and visual stimulation) followed by an interactive experience with biofeedback that provides a visual representation of the person’s heartbeat in real-time. For each of the conditions we assessed anxiety (state and trait), as well as other psychological variables pre- and post-experience. Additionally, we measured the heart rate variability (HRV) at baseline, during each stage of the experience as well as post intervention.

**Results:**

We collected valid data for a total of 68 individuals. Both groups were similar regarding mean age, sex, and occupation and had similar prior experience with mindfulness. There were no significant differences regarding prior state or trait anxiety between the groups. Analysis of the physiological variables showed that for both groups there was an increase in the parasympathetic activity after the multisensory experience, with small differences in the conditions of stimulation. We did not observe significant differences between the pre and post measures for state of test anxiety. The observed parasympathetic activity variations after both experiences compared with pre and post-surveys demonstrate the importance of physiological vs psychological inspection beyond the common human rational experience that is not always resonate with the body’s response and impacts the needed literacy to self-awareness of emotional well-being.

**Conclusion:**

Participants in both conditions could effectively connect with the experience, while achieving a physiological response different from their baseline state. The acceptance of the designed stimuli was very high, although more research is still needed to uncover its full potential. In sum, the design of multisensory experiences using technology to create an interactive connection with the sensory stimulus, is a promising field in mindfulness and especially in practices involving sensory awareness through the monitoring of parasympathetic activity as an inference indicator of the present-moment connection.

## 1. Introduction

In the current context of uncertainty and decreased reported states of wellbeing, part of a global landscape is experiencing unfavorable trends regarding inequality, fear and armed conflict. Hence, we present a multidisciplinary effort to enhance people’s wellbeing through a non-traditional approach to mindfulness.

We aimed to offer people a space to connect with the present moment, in a way in which everyday anxiety could be reduced, and awareness could be increased. Such a space could serve, not as a substitute for a formal mindfulness practice, but as a “teaser” of such a practice, while inviting users to further explore the connection with the present moment through their senses. The current research included the design and test of a *story setup* whereby naive participants were exposed to an experience that allowed mimicking the initial fragmented sensory exposure toward a mindfulness practice.

With the growing evidence of the effectiveness of mindfulness interventions (Carsley et al., 2018), and in an attempt to innovate in digital technologies, generating new spaces for the practice of mindfulness could be a relevant approach for understanding of its future applications. Interventions that train mindfulness have proven to increase physical and psychological well-being, independent of the use of technology during the experience (i.e., see Conley et al., 2016).

### 1.1. What is mindfulness?

Mindfulness can be described as “the awareness that emerges through paying attention on purpose, in the present moment, and non-judgmentally to the unfolding of experience moment by moment” (Kabat-Zinn, 2013, p.145). It can also be understood as an umbrella term that includes cognitive processes like attention, awareness, memory, and acceptance or discernment. This practice originates in the Buddhist tradition, in which the word mindfulness can be traced to “Pali,” a word in Cati language that means to recollect (Lukoff et al., 2020). Nowadays, the practice is based on a mix of traditional principles, contemporary practices, and neuroscience findings. For this work, we understand mindfulness as a state, not a trait (Davis and Hayes, 2011), and focus primarily on mindfulness-enhancing practices such as mindfulness meditation, applying one’s attention to breathing and bodily sensations (Davis and Hayes, 2011).

The following can be identified in the literature as benefits of mindfulness, categorized in three domains as proposed and presented by Davis and Hayes, (2011): (a) *affective benefits,* such as emotion regulation that manifests in a decrease in depressive or anxious symptoms and increase in working memory, particularly under stress as well as an increase in cognitive flexibility and attentional functioning; (b) *interpersonal benefits* such as an increased capability of expressing feelings in social situations, less emotional stress and an overall increase in relationship satisfaction; and (c) other *intrapersonal benefits* such as improved personal self-awareness in terms of morality, intuition and fear modulation, increased information processing speed, and neuroplasticity leading to altered brain structures.

### 1.2. Attention awareness

Attention awareness is a prerequisite for mindfulness and it can be thought of as a first step to mindfulness. It manifests as soon as an individual starts (1) noticing what is there (i.e., a thought, a feeling); can (2) name it and start indulging into more complex stages, (3) can let go, and feel (4) self-compassion, and/or (5) expanding (Strosahl et al., 2015).

But mindfulness in its most simple form also refers to some form of attention training, which can be done through one of mindfulness’ main principles: the “beginner’s mind.” This idea comes from the supposition that habituation to the everyday environment is a barrier to appreciating and seeing everything with curiosity. The beginner’s mind proposes the cultivation of the ability to see everything as if it were the first time (Kabat-Zinn, 2013) so that the ability to notice and pay attention to the present moment and connect to the surroundings even in everyday activities is reached. Exercises for this can be done with the simplest and most mundane tasks such as drinking coffee, eating, walking, and taking a shower or going to work and for many of these exercises, the breath serves as an anchor to attention training. Nonetheless, this training or practice can also be achieved by focusing on the senses and sensorial input of all kinds.

### 1.3. Mindfulness and technology

There is an apparent paradox when relating mindfulness to technology, where some experts argue that mindfulness not only preserves its positive effect on wellbeing when mediated by technology, for example, in the workplace (Wrede et al., 2023), but it can even make the practice more motivating and increase adherence (Wrede et al., 2023). Indeed, gamification and digitalization have expanded the reach of mindfulness.

However, gamification and digitalization also lead to an increasing ethical responsibility to design experiences according to the mindfulness principles and avoid designing technology-empowered experiences that can lead to comparison, judgment, competition, and other potentially detrimental applications that can blur mindfulness and misdirect personal growth (Lukoff et al., 2020). Here, it is also important to take into account that the individual relies on technology for accessing his or her senses (Wrede et al., 2023) and the consequences this may have for future practice.

Nevertheless, technological applications have been used for the development of mindfulness abilities with some early indications of support even for mediating interventions for depression, stress, and anxiety (Fish et al., 2016). Smartphone applications are the most accessible form, with most of them focusing on relaxation and stress reduction as outcomes, most of the time providing some evidence consistent with well-being improvements (Lukoff et al., 2020).

Other developments rely on more complex systems, such as objects that react to physiological signals to accompany mindfulness practices (Zhu et al., 2017; Vianello et al., 2019), EEG systems (Krigolson et al., 2017), virtual reality (VR) experiences (Kosunen et al., 2016), and mixed reality games (Roo et al., 2017). For many of these applications, authors find effects on relaxation (Shaw et al., 2011; Kosunen et al., 2016). Based on the aforementioned, our work is a part of the line of exploratory research that studies the role, mechanisms and potential deployment of complex sensory experiences mediated by technology that can foster mindful practices.

### 1.4. Multisensory experiences

Multisensory experiences can be described as “impressions formed by specific events, whose sensory elements have been carefully crafted by someone” (Velasco and Obrist, 2020). These experiences are usually designed with a specific purpose and, in this case, focusing on mindfulness experiences through a beginner’s mind practice. Multisensory experiences offer a milieu of different approaches on how to use mindfulness tools in innovative intuitive approaches. To guide the user to relaxation through sensory stimulation, most experiences have the intention to reach parasympathetic activation. For observing this, the use of physiological signals of biofeedback provides a widely accepted proxy measurement.

Other approaches that enhance the mindfulness experiences through multisensory stimulation with technology have also been taken, such as absence sensory stimulation (Vidyarthi and Riecke, 2014), stimulation that responds to physiological signals with abstract (Briant et al., 2015) or metaphorical stimulation (Yu et al., 2017).

For example, the Stress Tree is a project in which a virtual tree metaphorically represents the user’s health, the tree grows and changes its color depending on HRV data. Participants in this study reported that the relation with nature and health was intuitive and relaxing (Yu et al., 2017). Meanwhile, the “Meditation Chamber” (Shaw et al., 2011) is a VR experience in which a difference in relaxation after the practice was observed, especially for those with no prior experience with meditation. This effect was also confirmed in the VR experience RelaWorld (Kosunen et al., 2016).

Biofeedback has also been applied in multisensory experiences and virtual reality. For instance, it was found that mounted displays with neurofeedback inserted in a virtual reality context had higher levels of presence and lower negative emotions than other methods (Kosunen et al., 2016). Here, novice users achieved deeper relaxation and deeper levels of meditation than similar users without the device. In addition, indicators such as heart rate variability during various types of meditation practices have been reported as criteria to promote consciousness (Wu and Lo, 2008). The variability could also be explored to study the self-consciousness to the senses that focus the mind and body into the present-moment and it is a motivation for our pilot study.

## 2. Materials and methods

### 2.1. Overview of the current study

This exploratory study integrates multisensory experiences and mindfulness, where the space itself gives feedback to the participant and narrates the metaphor of growing nature as a way to connect with the surroundings. Here, the participants get immersed into a story in which each of their senses is first fractionated to work with the beginner’s mind principles.

For the design of the study setup, we used the beginner’s mind principle as a framework to create the narrative in which the senses were fractionated to create curiosity and facilitate the focus of attention on each of the senses one by one: subsequently stimulating smell, sound, sight, and finally engaging the participant into an interactive multisensory environment with integrated sensory stimuli. The main goal was for the participant to “stay” in the present by focusing on the senses. The basis for this narrative was the mindfulness meditation of eating a raisin (Stahl and Goldstein, 2019): a practice that consists of experiencing present moment-awareness in which the attention on the senses is separated.

For the setup, the concept of coherence in design was fully incorporated as well as the metaphors and mechanisms of technology aligned with its underlying principles (Lukoff et al., 2020). Eventually, all of the senses are integrated in an interactive multisensory experience that was conceived to generate feelings of acceptance and connection with nature.

Prior to finalizing the design of the study, we obtained the approval of the committee for ethics in research of Universidad de los Andes (file 1,445 of 2021), reviewed all relevant risks for the participants and developed a comprehensive informed consent process and form.

### 2.2. Design and procedure

There were two conditions—control and treatment, both preceded by a baseline assessment of HRV (4 min) and a post experience assessment (4 min). Treatment condition consisted of the following activities, each 4 min long. The total duration of the experiment was 24 min.

#### 2.2.1. Main experience timeline

**Figure 1:**
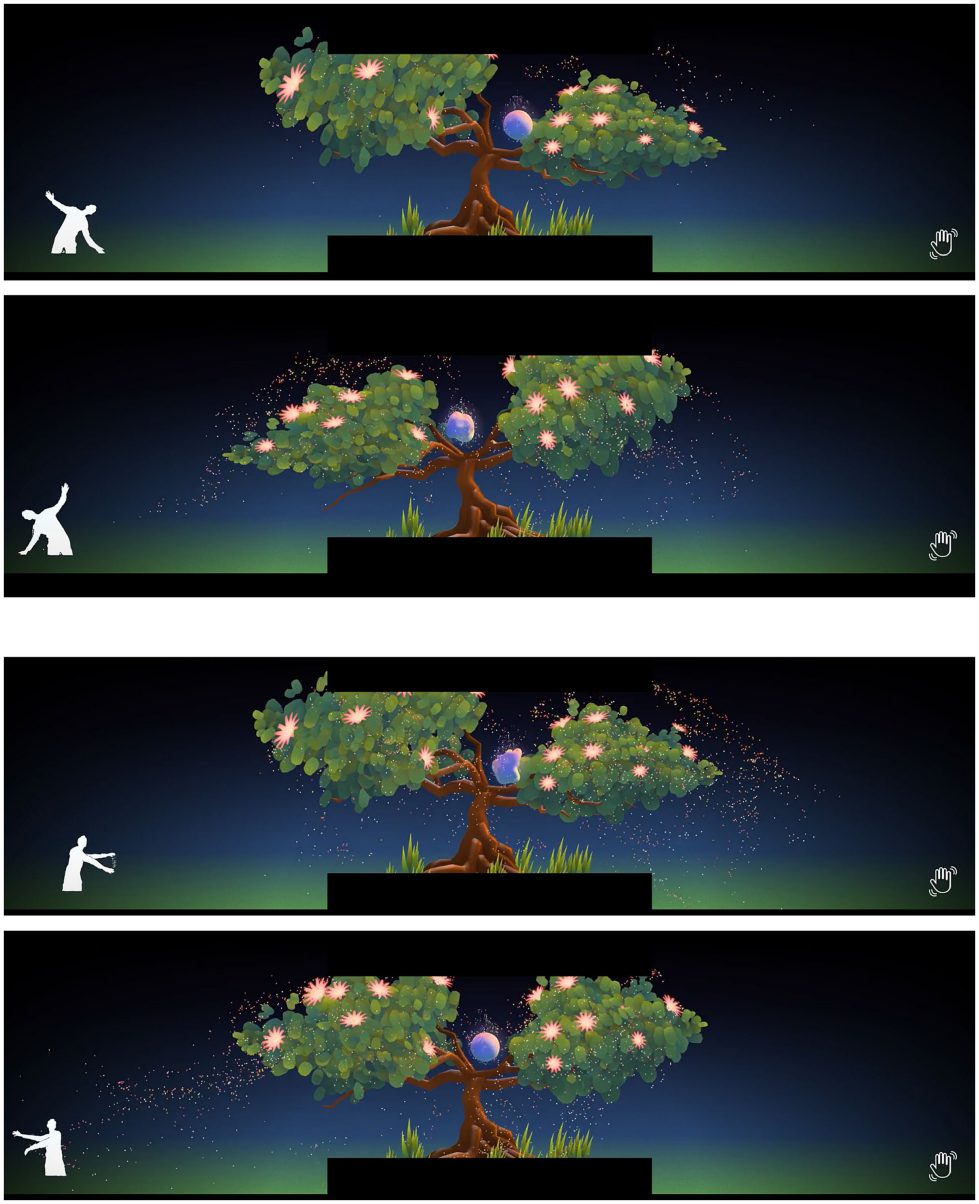
Four examples of interactivity, where the tree is bending and moving its branches according to the participant’s movement, and pollen particles are affected by the wind generated by the participant.

A. Participants entered the darkened room in which the experience took place. Informed consent was obtained and instructions were presented both about the purpose of the study and the use of biofeedback. Participants were also instructed to concentrate on their breathing and let thoughts pass by. During this introduction, the baseline assessment was performed (4 min).
B. Participants received the instruction to focus their attention on their sense of smell and were exposed to two fragrances coherent with the idea of nature: “Outdoors” and “Ylang” both from Signature Olfactive® (4 min).
C. An audio soundscape was then played, which included sounds of the four basic forces of nature (earth, water, wind, and fire). Afterward, there was silence and then “life began” with bird sounds, insect sounds, and instrumental piano music at the climax of this part of the experience. Slowly sounds faded again into silence (4 min).
D. A video, without sound, was then projected on three walls (front, left-, and right-hand side). This video showed an abstract expression of the cycle of life and the shapes and colors (4 min).
E. Finally, an interactive audiovisual experience connected the user’s heart rate response to a dynamic life cycle of a tree. It started with an image of a tree that grew from a seed, flourished and finally died, which responded to the movements of the participant and that had at its core a visual representation of the participant’s heart beating in real time (4 min; see Figure 1).
F. The post experience assessment was carried out at the end of the intervention (4 min).

#### 2.2.2. Control condition

For the control condition, we designed an experience that would emulate an audio guided formal (sitting) meditation practice focusing on three senses (smell, sound, and other sensations), that included the same time frames of the experimental conditions (4 min each), also included the HRV monitoring but in which there were no stimuli presented intentionally to the participant. First, the person was instructed to shut their eyes and focus on their smell, then on the surrounding sounds, and finally on their kinesthetic perceptions, while all the time maintaining a general focus on breathing. Participants were also instructed that, in case their mind wandered, they should gently let their thoughts or feelings go, noting them, and then returning to the breath or the sense they were focusing their attention on. The baseline and post-experience assessments of HRV (4 min each) were also performed. Hence the control condition took participants 20 min to complete.

### 2.3. Physiological signal acquisition and processing

The physiological changes throughout the experience were assessed in a way that allowed analyzing the effect that each of the experimental conditions had on the participants, independently. For this, we chose to analyze cardiac activity. More specifically, we recorded the electrocardiogram (ECG) of each participant using the Polar® H10 sensor chest strap and a custom developed software.

Cardiac activity is predominantly controlled by two branches of the autonomic nervous system (ANS)—the sympathetic and the parasympathetic nervous systems. Sympathetic activation is usually associated with “flight-or-fight” reactions that send commands for an increase in the heart rate (HR), although these have also been recently linked with states of mental stress (Castaldo et al., 2015). Conversely, the parasympathetic system is activated predominantly during a variety of resting conditions; i.e., its effect on the cardiac systems includes reducing heart rate and blood pressure, reducing respiratory rate, and conserving energy through relaxation and rest (Valenza et al., 2018). Thus, by measuring the activity of these systems one can better understand the state of the participant during an experience (i.e., relaxation through sensory stimulation).

Autonomic nervous system activity can be obtained by performing a heart rate variability (HRV) analysis. As it was first proposed by Akselrod et al. (1981), it measures the fluctuations around the mean of the heart rate (Akselrod et al., 1981). The basic unit for the analysis of all HRV metrics is the sequence of time intervals between heart beats, where such a time series is called inter beat intervals (IBIs). Sympathetic and parasympathetic activation can alter the IBIs; for instance, by raising the HR these become shorter.

However, the activation dynamics of the two branches of the ANS is very much differentiated. Parasympathetic influences are pervasive over the high-frequency range of the heart rate power spectrum, whereas the sympathetic influences stop at about 0.15 Hz (Thayer, 2020). Given these dynamics, there are several metrics that can be derived from the IBI that can help analyze the influence of the ANS branches in cardiac activity. One intuitive way of extracting this information is to look at the power spectrum of time series. By differentiating between high-frequency (HF, 0.15–0.40 Hz) and low-frequency (LF, 0.01–0.15 Hz) one can obtain the relative contribution of the systems to cardiac activity. A common proxy for assessing this is by calculating the LF/HF ratio (Kleiger et al., 2005). Nonetheless, spectral analysis is not the only way of obtaining information regarding ANS activation as time analysis metrics are also widely used. For instance, it has been shown that the root mean square of successive RR differences (RMSSD), which can also be obtained through IBIs, is strongly correlated to parasympathetic activation (Valenza et al., 2018).

### 2.4. Interactivity and immersion of setup

From the technical design standpoint, we required an “all-in-one” development platform that could handle a timeline system with event triggers, data streaming, video and audio playback, projection mapping, and sensor integration. With all of this in mind, we decided to use Touchdesigner^Ⓡ^ as the backbone for the experience, as it allows communication via Open Sound Control (OSC) and enables the use of the Kinect SDK needed to capture the users position and provide interactive feedback. However, in order to sync the data acquisition systems and the sensory stimulation we created a custom software that triggered each one of the sections automatically through OSC and handled the heart activity data acquisition.

#### 2.4.1. Design of video section

The design of the video section was inspired by immersive experiences and projection mapping artworks. Specifically, we immersed the participants in an abstract representation of the cycle of life, conceptually represented as a “zoom-out” from the microscopic world all the way to human scale. The video plays with the space of the projection, guiding the participant’s gaze with content on surrounding walls until it completely fills the room with light and color.

#### 2.4.2. Design of sound section (soundscape)

The soundscape was designed with the same principles of semantic congruence among the stimuli (i.e., Spence, 2007; Reinoso-Carvalho et al., 2019) and was co-designed by Hooked Music® Hamburg, Germany. It started with the sounds of the elements, followed by silence, and then life blossomed with sounds of nature, insects, and bird chirps. For a climax of the experience, a composition with piano accords was included until silence transitioned to the end of the soundscape.

#### 2.4.3. Design of interaction

The interactive part of the experience consisted of a representation of the life cycle of a tree, from seed until its death. During this journey, the participant’s movement and heart rate signals affected several elements of the artwork.

In the seed stage, the seed followed the participant through the space. Once the tree started to sprout, the participant was presented with an abstract heart that had a unique color palette for each person. The frequency of the beating of the virtual heart was synchronized with the real-time data acquired by the heart sensor of the individual.

Also, the tree reacted to the participant’s movement as if its two main branches were the person’s arms. Other subtle interactions were designed, such as the wind direction which depended on where the arms of the participant were pointing toward (see Figure 1 and access here a link to the video) and also certain sounds like those of the tree growing and moving.

### 2.5. Participants

Participants were contacted through campus ads and word of mouth, around the campus of Universidad de los Andes (Bogotá, Colombia) and both conditions took place in the same room under similar darkness conditions in order to minimize de influence of environmental factors on the physiological data obtained. Participants included students, professors, administrative personnel and members of the university community. They were assigned to the control or the treatment condition, depending on which one was running at the time they signed in, and were asked to pre-book a time slot for the experience. “No show” participants did not differ from those that attained with regard to their socio-demographic characteristics. We collected data from 88 participants but only included the 68 full data sets (pre-post questionaries, physiological measures and a fully functioning experience) of the control (*n* = 29) and treatment condition (*n* = 39) in subsequent analysis. See Table 1 for more details on participants and distribution.

**Table 1:** Basic sample characteristics.^*^

*Condition*	*Control*	*Treatment*
State anxiety (Pre)	48.10 (12.0)	49.97(10.30)
Trait anxiety	37.44 (10.05)	37.56(12.49)
Age	26.97 (9.56)	25.85(8.98)
SES	3.90 (1.29)	3.95 (1.43)
Mindfulness prior experience	1.55 (0.57)	1.42 (0.63)
Gender (Female%)	61%	37%

^*^Numbers in brackets are SDs.

### 2.6. Measures

#### 2.6.1. State–trait anxiety inventory

The State–Trait Anxiety Inventory (STAI) aims to evaluate anxiety as an individual difference in anxiety-proneness and as a day-to-day manifestation (Spielberger et al., 1971). This inventory consists of two 20-item scales, one for the individual general presence of anxiety (factor A-trait) and another for the level of anxiety present at the moment (A-State).

The STAI was initially translated into Spanish by Spielberger et al., 1971. There have been various translations and validations in Latin American populations as well (Silva et al., 2016; Villarreal Sánchez et al., 2019). For this study, the scale showed excellent reliability (state: α = 0.95, ω = 0.94; trait: α = 0.95, ω = 0.94).

We assessed trait anxiety only at the beginning and to control for more intrinsic aspects related to the benefits of the experience (e.g., if anxiety is a more stable condition in time), but the state scale was used both as a pre and post experience measure.

#### 2.6.2. Connectedness to nature scale

The connectedness to nature scale (CNS) is an instrument designed to measure “individuals’ experiential sense of oneness with the natural world” (Mayer and Frantz, 2004, p.504). A Spanish version of this scale was used to understand if an immersive experience with nature could increase the sense of connectedness at the moment. This one factor scale consists of 13 items. Reliability estimates for the current study were very high (*α =* 0.82, *ω =* 0.84).

There is strong evidence that this scale is valid and reliable (Mayer and Frantz, 2004). The Spanish version has been shown to have acceptable values for internal consistency (Olivos et al., 2011). Moreover, these scale scores are correlated with biospheres’ concerns and negatively related to egoistic orientation (Mayer and Frantz, 2004; Schultz et al., 2004).

We also asked participants to fill out the CNS both before and after the experience.

#### 2.6.3. Mindfulness attention and awareness scale

The Mindfulness Attention and Awareness Scale (MAAS) is focused on measuring the prevalence or absence of attention and awareness by evaluating differences in the frequency in which states of Mindfulness are experienced (Brown & Ryan, 2003). Therefore, it asks about experiences, sensations, emotions, and level of attention in daily life and is related to Mindfulness more as a trait than a current state. It is composed of 15 items that belong to the same factor. The items follow a six-point Likert scale, where 1 = Almost always and 6 = Almost never, is the first scale in the questionnaire. We used this questionnaire to control for Mindfulness-related attitudes in daily life, acquired through prior practice and that could confound with the benefit that this particular practice could have on an individual.

When used in a sample of Colombian university students the MAAS had an excellent internal consistency (*α* = 0.92), all the results of expected correlations with other instruments were established in the expected direction and discriminatory validity was found (Ruiz et al., 2016).

#### 2.6.4. Toronto mindfulness scale

The Toronto Mindfulness Scale (TMS) was chosen for the post-experience assessment, as it addresses mindfulness as a state-like quality measuring the subjective state at the moment, and not as a trait (Lau et al., 2006). This 13-item scale is made out of two factors, curiosity, and decentering. This scale was used to measure the mindfulness state of the participant after taking part in the multisensory (or control) experience.

This scale has shown significant and reliable face value results (Gherardi-Donato et al., 2020) and internal consistency with Cronbach’s alphas on both factors, curiosity (ranging from 0.86 to 0.91) and decentering (0.85 to 0.87; Park et al., 2013). For this research, we applied a Spanish translation and back translation process, as well as a cognitive pre-test to guarantee the instrument’s validity. For the current study, both subscales exhibited good consistency scores (Curiosity α = 0.80, ω = 0.81; decentering α = 0.74, ω = 0.74).

#### 2.6.5. Qualitative assessment

After the experiment, as a complementary assessment, the participants were invited to qualitatively reflect on the experience that they just had via ratings of the experience, referral to family and friends, ratings of satisfaction with the single conditions as well as open-ended questions. The researchers in charge of this assessment compiled the most relevant comments, which were used as a kind of validation of the principal quantitative results mentioned above.

#### 2.6.6. Heart rate variability

We used two of the representative parameters of HRV as methods of measuring HRV (root mean square of the squared differences of the RR intervals of successive heartbeats—RMSSD—; and the standard deviation of the RR intervals—SDNN). SDNN is a general measure of HRV, while RMSSD indicates parasympathetic activity in particular. As previously mentioned, we also assessed the Lf/Hf ratio (Shaffer and Ginsberg, 2017).

### 2.7. Data analysis

All analyses were conducted using R statistical software (R Core Team, 2022). For the assessment of differences between groups at baseline, we performed pairwise comparisons Bonferroni adjusted. For the assessment of the effect of time and differences between groups, we fitted a mixed model...with an error term for participants—to account for the repeated measures nature of the data—, with the *lme4* library (Bates et al., 2015) was used. We also calculated a change index by subtracting the baseline from the final scores and used it as our key dependent variable for a series of generalized linear models. Then, pairwise contrasts using Bonferroni-corrected *post hoc* tests were conducted with the Kenward Roger method for calculating degrees of freedom. Two-sided values of *p* <  0.05 were considered statistically significant (95% confidence). Qualitative topics were analyzed with Qualtrics.

## 3. Results

Both groups were similar with regard to socio-demographic and psychological variables, with no statistically significant differences among them. There were also no differences at baseline for any of our three key measures or the heart rate variability (Lf/Hf [*t*(63.48) *=* −0.15, *p =* 0.87], RMSSD [*t*(59.95) *=* −0.25, *p =* 0.80], and SDNN [*t*(57.04) *=* −0.64, *p =* 0.23]). Groups were also similar in other characteristics at baseline such as coffee consumption (38 vs. 42% of participants reported having a coffee earlier in control and intervention conditions, respectively), self-reported hours of sleep and quality of sleep [5.45 (control) vs. 5.3 (intervention) hours, with a mean of 11.5 in a scale from 1 (poor) to 16 (excellent) in both conditions], and prior experience with mindfulness practice (50 and 65% of people in control and intervention conditions, respectively, reported having some experience with mindfulness practices).

Regarding our psychological variables, respondents in both conditions benefited in terms of their state-anxiety (reporting significantly lower levels after the experience than before) and their reported connection to nature increased significantly after the experiences, when compared to before. This was evident for both conditions (see Table 2 for details). Interestingly, if calculated by a cut-off score of 44 points (as proposed by Ercan et al., 2015), 69.1% of women and 67.6% of men participating in both conditions would have reported a clinically significant anxiety at the beginning of the experience, which would have reduced to 49.5% women and 47.1% men at the end.

**Table 2:** Synthesis of the descriptive results on relevant scales.

*Measure*	*Control*	*Treatment*
State of anxiety (Pre)	48.10 (12.0)	49.97(10.30)
State of anxiety (Post)	43.13 (5.13)^**^	44.79 (4.16)^**^
TMS curiosity	20.10 (6.55)	22.20 (5.03)
TMS decentering	22.30 (6.55)	24.69 (4.43)
CNS Pre	3.44 (0.46)	3.48 (0.49)
CNS Post	3.68 (0.52)^**^	3.63 (0.70)^**^
MAAS	53.13 (13.02)	53.53 (13.84)

^**^STAI State Anxiety significant to *p* > 0.001 for pre/post. ^**^CNS ANOVA repeated measures significant to *p* > 0.001 for pre/post *d* = 0.40.

We performed a 2 (condition) × 2 (anxiety: pre post) ANOVA to examine differences in anxiety, before and after the experience. This analysis revealed a main effect of anxiety but not of condition, meaning that anxiety did decrease similarly for both treatment and control conditions. *Post-hoc* tests revealed though that the difference in the decrease was slightly larger for the treatment condition [5.18, *t*(38) *=* 3.35, *p =* 0.001] than for the control condition [4.96, *t*(28) *=* 2.52, *p =* 0.017], even though in both conditions state of anxiety decreased significantly.

Regarding the “effectiveness” of the intervention in terms of mindfulness, assessed through the TMS, participants in both conditions scored higher on the decentering, than on the curiosity scale after the experience.

Table 3 contains the descriptive statistics by condition and measure. We tested for experimental differences by fitting mixed effect models with experimental condition and measurement modality as predictors for each measure (see Appendix for complete results). In general, we did not find any statistically significant differences between the experimental and control group regarding the response of the parasympathetic system of the participants to the complete experience. Even though there was a significant interaction between the group and the final measurement ( β = 15.48, *p =* 0.043), the *post-hoc* analyses did not reveal any statistically significant differences.

**Table 3:** HRV measures for control and treatment conditions.

		LF/HF				RMSSD				SDNN			
Condition	Modality	Mean	SD	Min	Max	Mean	SD	Min	Max	Mean	SD	Min	Max
Control	Sound	5.04	6.65	0.14	27.29	55.72	37.59	12.55	165.26	77.65	34.00	27.10	181.23
	Baseline	3.50	3.52	0.18	13.25	51.17	52.96	7.55	253.65	67.49	43.86	14.99	219.18
	Final	1.87	1.86	0.16	8.39	56.96	45.07	11.08	238.27	67.75	35.22	23.68	172.20
	Interactive	3.30	3.49	0.18	16.00	53.96	46.92	11.43	239.31	79.49	32.40	25.21	169.17
	Smell	3.17	3.39	0.24	12.04	57.24	43.91	11.49	193.87	76.94	46.63	21.12	217.79
	Visual	3.49	5.86	0.16	27.94	54.66	41.26	11.37	204.37	74.19	33.05	24.54	168.67
Treatment	Sound	4.88	3.71	0.36	18.13	47.54	38.91	9.73	186.71	73.98	36.76	31.63	199.98
	Baseline	3.63	3.90	0.36	14.88	54.46	52.14	12.12	275.86	74.11	39.80	27.38	226.42
	Final	3.46	4.77	0.15	23.20	61.15	48.37	10.49	216.57	79.56	40.32	23.07	191.01
	Interact	4.50	4.47	0.43	21.37	49.91	38.71	12.08	187.91	78.36	33.24	30.15	164.86
	Smell	5.53	5.29	0.17	20.69	51.18	46.30	11.40	216.31	76.47	38.09	27.10	213.39
	Visual	4.38	4.00	0.25	18.99	47.79	42.94	10.17	221.14	69.31	38.12	25.43	184.45

Moreover, the different senses stimulated in the different moments of the experience did prompt different effects on participants of both groups. We found significant interactions between the olfactory stimulation and condition for Lf/Hf ( β = 2.52, *p =* 0.029). *Post-hoc* analysis revealed a significant decrease for the treatment group [*t*(202) *=* −2.18, *p* < 0.05]. The latter means that the olfactory stimulation yielded better results in terms of parasympathetic activation in the treatment group than in the control group.

To further explore possible differences between the treatment and the control group, and to verify for individual variations in heart rate variability prior to the experience, we calculated a change index by subtracting the final measurement from the initial baseline, and used it as a dependent variable for a set of exploratory generalized linear models. This led us to assess whether the Lf/Hf and RMSSD measures were predicted by differences between treatment and control groups, which was indeed the case but only when an interaction with the score for trait Mindfulness (MAAS score) was included (see Appendix). However, *post-hoc* analyses did not reveal any further differences.

Note the participants in the treatment condition reported via questionnaire that the interactive experience helped them relax more compared to the control group [*M_control_* = 2.89, *M_treat_ = 3.25, t* (57.93) *=* −2.08, *p* < 0.05] and also increased attention across visual [*M_control_* = 6.68, *M_treat_ = 8.01, t* (54.34) *=* −2.07, *p* < 0.05], movement [*M_control_* = 7.24, *M_treat_ = 8.52, t* (49.18) *=* −2.13, *p* < 0.05], and auditory [*M_control_* = 6.68, *M_treat_ = 8.40, t* (56.8) *=* −2.83, *p* < 0.01] stimulation.

Experience evaluation was also associated with some key measures. For instance, participants’ acknowledgment that the interactive experience helped them relax was significantly correlated with their heart rate variability measures: RMSSD [*r*(67) = 0.27, *p* < 0.05] and SDNN [*r*(67) = 0.28, *p* < 0.05]. The RMSSD was also positively correlated with a desire to have more similar interactive experiences [*r*(66) = 0.24, *p* < 0.05].

Qualitative analysis of the responses of both groups showed that there were common themes among them, like the appreciation of sensory experiences, the benefits of mindfulness and relaxation and the importance of spaces on campus devoted to wellbeing, as well as the overall impact of the experience. One participant stated: “this was a space for myself”. Nonetheless, participants of the treatment condition reported a stronger emphasis on the visual and auditory elements, highlighted the benefits of such a technology—driven experience (for example mentioned their appreciation of the tree’s movement interactivity) and reported a stronger connection to nature, for example one participant reported: “the fact that I had an individual space to connect with my senses. I loved the interactive part”.

## 4. Discussion

In this multidisciplinary exploratory assessment, we analyzed whether a mindfulness practice could be compared to a multisensory experience design in terms of its impact on the “state” of mindfulness of an individual. For this, we piloted and designed a study involving two conditions: First a guided mindfulness practice based on the senses as an anchor to the present moment, using audio instruction only (namely control). Second, an experience of mindfulness practice with successive multisensory stimulation less formal in the instruction but with an anchor on the senses and backed up by the principles of the beginner’s mind.

The first finding in our study is that we worked with two groups that were similar about the most relevant variables, such as age, sex, and their previous mindfulness practice, and that after both conditions we could observe a parasympathetic response in the direction we expected in both groups. This means that the participants obtained a “relaxation” effect in both conditions. However, when it comes to the qualitative assessment (open-ended question), the participants consistently reported feeling more relaxed under the multisensory experience. Participants also reported being keen to further use such kinds of technological aids in the future and wanted to obtain such offers permanently in their daily lives on site.

Our results also showed that the control condition yielded promising results in terms of parasympathetic activation and relaxed states of mind. This could be related to the fact that even a short period dedicated to pausing and concentrating on the present moment can have a benevolent direct effect on the nervous system. Hence, the opportunity to use simple low-tech methods to induce the state of mindfulness is encouraging as it implies that the democratization of such solutions to the benefit of mental and physical health are accessible to all, irrespective of their socioeconomic realities.

As to the effectiveness in mindfulness training, our results showed that participants in both conditions reported higher values in decentering than in curiosity, which aligns to previous findings relating technology to mindfulness training in their case through virtual reality, and found similar results (Chandrasiri et al., 2020) ().

Participants of both conditions reported having enjoyed the experience, wanting to use similar experiences in the future and highly recommending this type of experience to their friends and relatives. The open-ended questions also yielded some contradictory results about how to enhance the experience, indicating the highly subjective sensations across all stimuli. For example, some participants wanted stronger olfactory stimulation, while others wanted it to be less intense.

Overall, one of the most important findings of this study is the alarming level of anxiety that was reported by our participants in both conditions prior to the study. More than two-thirds of all individuals reported clinically relevant anxious states, both among men and women. This should be a warning sign and provides further evidence about the mental-health crisis much described after the pandemic in educational settings.

### 4.1. Implications and future investigations

After experiencing the study, the participants mostly reported the need for more spaces like this in their lives. On the other hand, however, prior to joining the study, most people were not as open to allocating time for their wellbeing. Such contradiction somehow aligns with the often-described discrepancy between the knowledge a person has about their habits and health, and the actual behavior in real life, where opposing motivations tend to collide. Nonetheless, for many of our participants, the sheer knowledge that a person can be mindful during everyday chores was enlightening and enriching. The observed parasympathetic activity variations after both experiences compared with pre and post-surveys demonstrate the importance of physiological vs psychological inspection beyond the common human rational experience that is not always resonate with the body’s response and impacts the needed literacy to self-awareness of emotional well-being.

Moreover, one of the advantages of the method presented here is the affordability of the equipment (using regular sports wearables, such as the chest strap) which could make further research on interventions similar to this one rather feasible. Indeed, technology-driven resources could be employed to further engage mindfulness-promoting interventions.

Note that the state of mindfulness seems to be a deeply individual experience that is optimally triggered and maintained through a different combination and intensity of customized stimuli. Future work can highlight how the individual experience of mindfulness can be successfully established and perfectioned and the role of technology in this process. In fact, many recent technology-based experiences claim that a person can obtain all the evident benefits of mindfulness meditation without long-term training (e.g., devices such as “sensate®” or mobile applications such as “BeLight®”).

The question of whether such one-time experiences could bring effects that are comparable to the effects of more traditional meditation or mindfulness practice that is built up with weeks, months, and years of training, remains open, where future similar studies could help address this question providing support for making mindfulness available to a wider audience (i.e., longitudinal assessments).

### 4.2. Limitations

The design of a space for a multisensory experience like the one we described here was a challenge in itself. For instance, obtaining institutional support for the allocation of space and resources was complicated. Moreover, the steady call for participants was also challenging, where the small convenience sample of our study is our main limitation.

Another limitation is the time to experiment. Perhaps a future replication of this study could find a way to optimize the experimental protocol to hopefully get more subjects to join the study, and to have a “true” control group which would not being exposed to mindfulness practices of any kind but going on “mindlessly” with other daily activities.

## Data availability statement

The raw data supporting the conclusions of this article will be made available by the authors upon request.

## Ethics statement

The studies involving humans were approved by Committee for Ethics in Research from Uniandes. The studies were conducted in accordance with the local legislation and institutional requirements. The participants provided their written informed consent to participate in this study.

## Author contributions

CF, AA, WJ-L, JB, DS, SH, FR-C, and VA have participated in the conception and writing of this manuscript. All authors contributed to the article and approved the submitted version.
