# Supplementary figures and images for: A multisensory mindfulness experience: exploring the promotion of sensory awareness as a mindfulness practice
Source: Front Psychol. 2023 Nov 9;14:1230832. doi: 10.3389/fpsyg.2023.1230832 (PMC10666737; doi:10.3389/fpsyg.2023.1230832)

Appendix

A.1 Mixed model results for each measure.


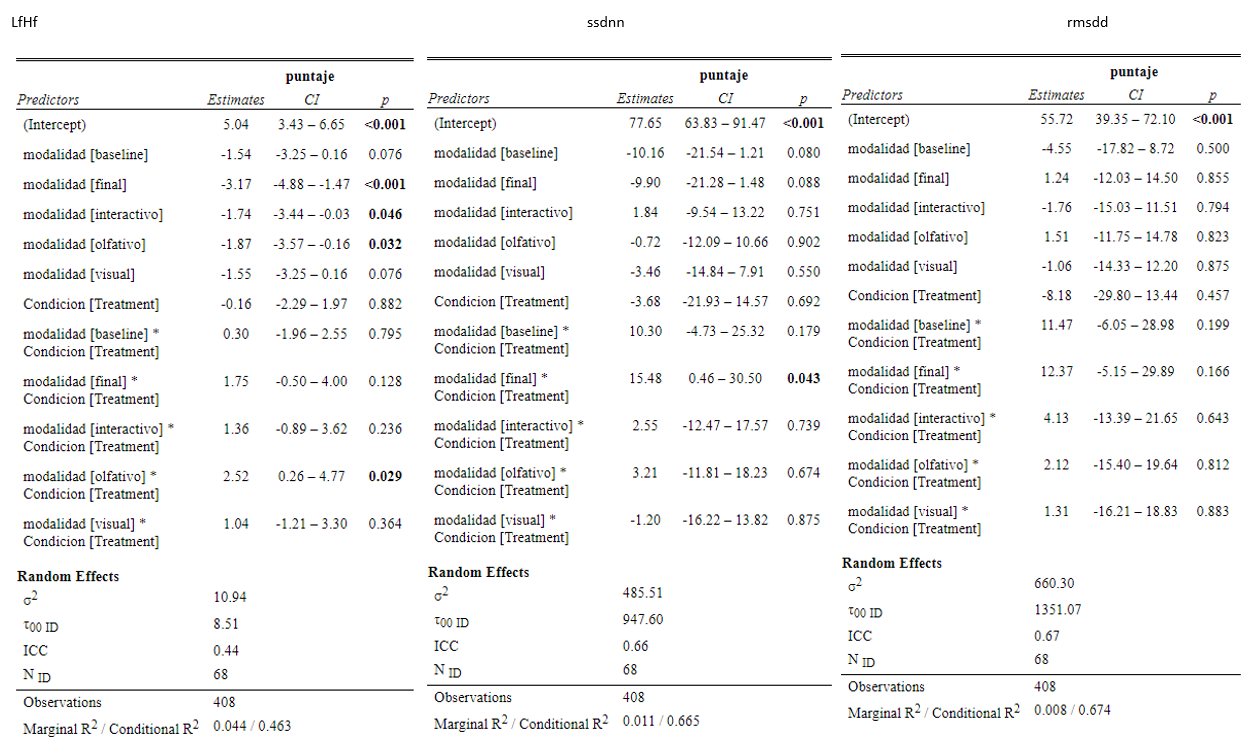


A.2 Exploratory GLMs by measure


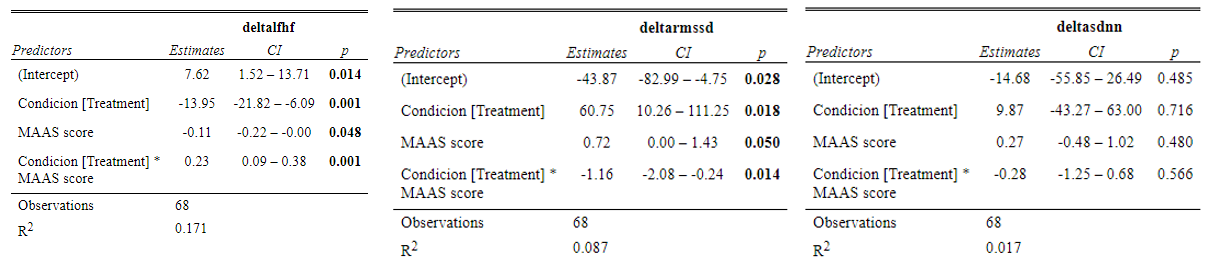

Supplement: Supplementary file 1 [file Table_1.docx]
